# Biomarker-Informed Machine Learning Model of Cognitive Fatigue from a Heart Rate Response Perspective

**DOI:** 10.3390/s21113843

**Published:** 2021-06-02

**Authors:** Kar Fye Alvin Lee, Woon-Seng Gan, Georgios Christopoulos

**Affiliations:** 1 Smart Nation Translational Laboratory, School of Electrical and Electronic Engineering, Nanyang Technological University, Singapore 639798, Singapore; ewsgan@ntu.edu.sg; 2 Decision, Environmental and Organizational Neuroscience Lab (DeonLab), Nanyang Business School, Nanyang Technological University, Singapore 639798, Singapore; CGeorgios@ntu.edu.sg

**Keywords:** cognitive fatigue, heart rate variability, biomarker, machine learning

## Abstract

Cognitive fatigue is a psychological state characterised by feelings of tiredness and impaired cognitive functioning arising from high cognitive demands. This paper examines the recent research progress on the assessment of cognitive fatigue and provides informed recommendations for future research. Traditionally, cognitive fatigue is introspectively assessed through self-report or objectively inferred from a decline in behavioural performance. However, more recently, researchers have attempted to explore the biological underpinnings of cognitive fatigue to understand and measure this phenomenon. In particular, there is evidence indicating that the imbalance between sympathetic and parasympathetic nervous activity appears to be a physiological correlate of cognitive fatigue. This imbalance has been indexed through various heart rate variability indices that have also been proposed as putative biomarkers of cognitive fatigue. Moreover, in contrast to traditional inferential methods, there is also a growing research interest in using data-driven approaches to assessing cognitive fatigue. The ubiquity of wearables with the capability to collect large amounts of physiological data appears to be a major facilitator in the growth of data-driven research in this area. Preliminary findings indicate that such large datasets can be used to accurately predict cognitive fatigue through various machine learning approaches. Overall, the potential of combining domain-specific knowledge gained from biomarker research with machine learning approaches should be further explored to build more robust predictive models of cognitive fatigue.

## 1. Introduction

Cognitive fatigue is a psychological state characterised by the subjective feelings of tiredness, insufficient energy, difficulty with concentration, and impaired ability to think [1,2]. This psychological state arises from cognitive “overloading” due to extended periods of sustained performance or cognitively demanding activities [1,3,4,5,6,7]. Attending meetings and report writing during work, as well as childcaring and meal prepping while at home, are examples of such activities. It should be noted that cognitive fatigue is not to be conflated with fatigue that arises from prolonged muscle activity (i.e., physical fatigue), emotional exhaustion (i.e., chronic fatigue) [8], sleep deprivation [9], or boredom [3]. Notably, cognitive fatigue has been demonstrated to have negative effects on executive functions, such as working memory, judgement, and attention [10,11,12,13]. Not surprisingly, cognitive fatigue increases the risk of accidents and errors in various mission-critical situations [14,15]. Hence, the ability to accurately assess and monitor cognitive fatigue levels during such situations is imperative in mitigating and minimising the risk of undesirable negative outcomes from occurring. While various methods have been developed to assess cognitive fatigue, these methods have their unique limitations and associated research gaps.

The present paper aims to examine the current research status on the assessment of cognitive fatigue and provide suggestions for prospective researchers regarding the measurement and analysis of cognitive fatigue, with emphasis on biomarker research and machine learning approaches. To this end, we searched for relevant research over the last three decades using various combinations of key terms, such as cognitive fatigue, mental fatigue, self-report, cognitive task, biomarker, heart rate variability (HRV), and machine learning via Onesearch and Google Scholar. Approximately 150 relevant published studies and reviews were identified and qualitatively assessed. 

The rest of the paper is organised as follows. First, we describe some traditional psychological assessments of cognitive fatigue. Thereafter, biomarker-based measures of cognitive fatigue are delineated. In particular, we evaluate the imbalance between sympathetic and parasympathetic nervous activity as a potential physiological correlate of cognitive fatigue, and how this can be indexed through HRV. Next, we present in detail recent data-driven approaches in predicting cognitive fatigue through machine learning. Last, we highlight some issues that should be considered when building models of cognitive fatigue. In sum, there are potential benefits in combining knowledge gained from biomarker research with data-driven approaches to build better predictive models of cognitive fatigue.

## 2. Traditional Psychological Assessments of Cognitive Fatigue

Within the psychological literature, several subjective assessments of cognitive fatigue have been developed and validated, such as the Mental Fatigue Scale [16], the Chalder Fatigue Scale [17], and the Fatigue State Questionnaire [18]. These self-assessments of cognitive fatigue are generally questionnaires that employ Likert scale ratings (e.g., “How tired does your mind feel right now? 1. Not at all 2. A little 3. Moderately 4. Very 5. Extremely”) [18]. The scores are then calculated to provide a general index of cognitive fatigue. Notably, such self-report methods presume that respondents have some level of insight or introspection into their cognitive states [19]. Indeed, while useful in gaining introspective knowledge of one’s psychological state, these self-report measures of cognitive fatigue require individuals to be self-aware of their fatigue levels [20]. Unfortunately, individuals often do not have an accurate judgment of their cognitive states [21]. Fatigued individuals have inconsistent self-awareness of their decline in performance [22]. Furthermore, the level of self-awareness of fatigue is moderated by varying working conditions [23]. People may not be able to appreciate how fatigued they are until it is actually “too late”, which can have devastating consequences in critical situations [24,25]. In addition, even brief questionnaires require disruption of current activities to allow time for assessment [26,27] and thus may not be suitable for use in situations that require continuous focus.

Alternatively, cognitive fatigue could also be objectively, but indirectly, inferred from a decrease in cognitive and behavioural performance over time [11,13,28,29,30]. These performance metrics, such as accuracy and reaction time, are typically measured in the context of computerised versions of cognitive tasks, such as the Stroop task [31] or the Simon task [32]. Previous research has demonstrated that these performance measures are associated with self-reported levels of cognitive fatigue [33,34]. For these performance measures to be used as indices of cognitive fatigue, it is assumed that the decrease in performance is a result of an individual’s impaired ability to maintain optimal task performance due to cognitive “overloading” [11]. However, these objective measures are often task-dependent, and thus the models based around these measures may have limited generalisability across different situations in predicting cognitive fatigue. For instance, Liu and colleagues [35] examining cognitive fatigue found that an increase in reaction time was observed only during an arithmetic task, whereas an increase in error rate was observed only during a switching task. Furthermore, in a low cognitive demanding task, such as a vigilance task, the authors found that neither reaction time nor accuracy could be used to index cognitive fatigue [35]. Interestingly, a recent study found that 16-min dual-tasks were more effective at inducing cognitive fatigue as compared to a 90-min single-task [36]. The same study also found that participants in the dual-task conditions had lower accuracy as compared to participants in the single-task condition [36]. Future research should examine whether using multitasking measures provide more reliable assessments of cognitive fatigue as compared to single-task measures. It is also important to note that these measures are only able to detect cognitive fatigue after a considerable decline in behavioural performance, which can often be detected too late in critical situations [37]. 

Despite their usefulness in furthering our theoretical understanding of cognitive fatigue, it appears that both self-assessments and task performance have limitations, in terms of subjectivity, disruptiveness, timeliness, and generalisability that are unsuitable for application beyond research settings. Subjectivity, in this paper, refers to whether the assessment is dependent on an individual’s self-evaluation and thus may be influenced by biases, such as socially desirable responses and lack of introspection. Disruptiveness refers to whether current activities must be stopped for a certain period of time for the administration of the assessment (e.g., when examining the effects of cognitive fatigue during driving, participants are required to stop driving for the assessment). Timeliness refers to whether the assessment is made in real-time or if there is a lead time between the point at which the assessment is made and the point at which the results are known. Finally, generalisability refers to whether the assessment can be extended to another individual, group, task, or situation. These four factors are important considerations when evaluating the suitability of applying these assessments in various settings. For instance, workplaces would prioritise subjectivity, disruptiveness, and timeliness over generalisability. A summary of these assessments is described in Table 1.

## 3. Autonomic Nervous System Biomarkers of Cognitive Fatigue

**Figure 1:**
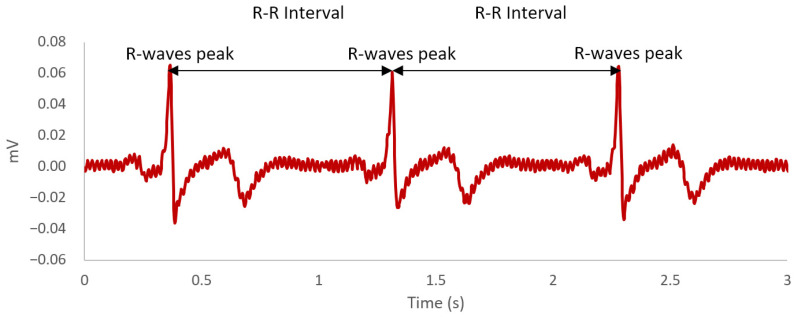
An example of a heartbeat waveform across a 3-second time window. The R-wave peaks are the most positive deflection observed in the waveform. The R-R intervals are the time difference between each R-wave peaks.

Over the last decade, there is a growing interest among researchers in identifying potential biological markers of cognitive fatigue [41]. A biological marker, more commonly referred to as biomarker, is defined as “a characteristic objectively measured and evaluated as an indicator of normal biological processes, pathogenic processes, or pharmacologic responses to a therapeutic intervention” [42] (p. 91). Indeed, there is an accumulation of empirical evidence within the literature suggesting that the autonomic nervous system is a physiological correlate of cognitive fatigue [43,44,45,46,47,48,49,50,51]. The autonomic nervous system as part of the peripheral nervous system consists of two components—sympathetic and parasympathetic [52]. The functional significance of the sympathetic nervous system is to prepare the body for physical demands by redirecting oxygen-rich blood to areas of the body where needed, whereas the parasympathetic nervous system is responsible for saving energy for future use as well as regulating bodily functions when the body is at rest [52]. Notably, the parasympathetic nervous system plays an inhibitory-disinhibitory role with the sympathetic nervous system, facilitating the returning of the body to calm states and mobilisation of energy, respectively [53]. Both the sympathetic and parasympathetic nervous systems are regulated by the preganglionic sympathetic and parasympathetic neurons in the central autonomic network [54]. These neurons are linked to the heart through the stellate ganglion and vagus nerve [54]. It is the interaction between sympathetic and parasympathetic neuronal outputs from the central autonomic network on the sino-atrial node of the heart that produces the phenomenon of complex variation in time intervals between heartbeats, which is more commonly known as HRV [55]. HRV is defined as the variation in R-R time intervals on the heartbeat waveform (see Figure 1). Each R-R time interval is measured as the time between each successive heartbeat, indicated by the R-wave peaks on the electrocardiogram [56].

Besides autonomic nervous regulation, the various components in the central autonomic network are also responsible for facilitating cognitive functions that are key for goal-oriented behaviour and behavioural adaptation [57,58]. Consistent with the overlap in neural structures, previous research has demonstrated a relationship between vagal tone and cognitive functioning, such as working memory and attention [59]. Extensive physiological research has provided supporting evidence for the validity of indexing different aspects of autonomic nervous activity through various HRV components (e.g., [60,61]). These HRV components are derived from various types of analysis, such as time-domain, frequency-domain, and non-linear analyses. Time-domain analysis, in particular, is a form of linear analysis that examines HRV across a specific period of time, whereas frequency-domain analysis (also another form of linear analysis) decomposes HRV signals into various frequency bands [62]. Time-domain analysis is generally more suitable for long-term recordings as it is less influenced by the instability of heart rate modulation, while frequency-domain analysis is more commonly used on short-term recordings due to easier physiological interpretation [61]. By contrast, non-linear analysis purports to quantify the dynamic nature of HRV and thus provides a more accurate representation of the complex interactions amongst various autonomic mechanisms underlying the cardiovascular system [60,62,63]. However, some would argue that it is more difficult to interpret as well as map fundamental autonomic mechanisms to non-linear components [64]. Overall, the different types of analysis purport to assess the linear and non-linear components of the HRV. Some of the more common indices of HRV are presented in Table 2.

In particular, frequency-domain components have been extensively examined within the cognitive fatigue literature. This is likely due to the easier physiological interpretation of frequency-domain components and time constraints on experimental designs limiting the collection of long-term recordings. For instance, previous research has demonstrated lower high-frequency power after a 30-min 2-back task [48,49], 64-min Multi-attribute Task Battery [44], 90-min vigilance task [45], and 2-h set-shifting task [50] and simple arithmetic task [51]. A more recent study has also demonstrated the decrease in high-frequency power after an 8-hour fatigue-inducing task, which consisted of multiple sets of advanced trail making tests, kana pick-out tests, and mirror drawing tests [47]. Given that high-frequency power has been hypothesised to reflect parasympathetic nervous activity [65,70,71], it appears that decreased parasympathetic nervous activity is involved in cognitive fatigue.

However, previous research has also demonstrated higher low-frequency power after the aforementioned 2-back task [49], Multi-attribute Task Battery [44], vigilance task [45], shifting task [50], and simple arithmetic task [51]. A similar effect was also observed in a 4-hour driving task [43] and, albeit marginal, a 140-min visual tracking task [46]. Given that low-frequency power is sensitive to both sympathetic and parasympathetic nervous activity [65,69,70,71,72], these findings indicate that the sympathetic nervous activity is also involved in cognitive fatigue.

In contrast to low-frequency power, the ratio of low- to high-frequency power arguably serves as a more precise indicator of the balance and interaction between the sympathetic nervous system and parasympathetic nervous system [70,73]. An increase in the ratio of low- to high-frequency power after the vigilance task [45], set-shifting task [50], simple arithmetic task [51], and the 8-hour fatigue-inducing task [47] has also been found in previous studies. While inconsistent and marginal, a similar effect has also been observed in the 2-back task [48,49]. Furthermore, Tanaka and colleagues [49] also demonstrated that greater sympathetic nervous activity was associated with greater levels of self-reported fatigue, while lesser parasympathetic nervous activity was associated with greater levels of fatigue. In addition, the predominance of the sympathetic nervous activity in the overall autonomic nervous system was also positively associated with self-rated fatigue levels [47,49]. Overall, the pattern of results suggests a sympathovagal imbalance with a shift towards sympathetic predominance may be linked to cognitive fatigue in the general population. The sympathovagal imbalance, or rather balance, can be broadly conceptualised as the dynamic influence of the sympathetic and parasympathetic nervous activity on one’s cardiac state [80]. Hence, it appears that there is empirical evidence satisfying the biomarker evaluation criteria of association [81,82], indicating that sympathovagal imbalance may be a putative physiological biomarker of cognitive fatigue.

## 4. Digital Biomarkers of Cognitive Fatigue through Wearables and Machine Learning

With the advent of affordable mobile phones and wearables, the large amount of data collected from these devices can provide extensive information regarding the user [83,84,85], including working professionals [86]. Consequentially, using data-driven approaches, such as machine learning, to process these large datasets appear to be a promising avenue in predicting one’s current psychological states [87]. Machine learning is a field within artificial intelligence broadly defined as an algorithmic approach that detects patterns through automation and optimisation, with minimal user input, to make predictions or decisions [88,89]. In practice, machine learning allows researchers to build computational models from large datasets that can learn, classify, predict, and improve through training [90,91,92].

When developing a machine learning model, a large dataset is typically divided into three subcategories—training, validation, and test datasets [93]. The training dataset is used for model fitting [93,94]. By contrast, the validation dataset is used to provide prediction error estimates of the fitted models during model selection [93]. In addition, the validation dataset is also used to make tuning adjustments to the parameters for further optimisation of the model [94]. Last, the test dataset is used only once after the training and validation phase to provide an unbiased assessment of the prediction error of the final model [93,94]. Ideally, a given dataset would be split into these three subsets for building, optimising, and evaluating a machine learning model.

Throughout the model building process, the models are evaluated with multiple performance metrics [95,96,97,98,99,100] (refer to Table 3). Accuracy is one of the key performance metrics of a robust machine learning model. In the context of a binary classification model, accuracy is calculated by the proportion of correct predictions divided by the total number of predictions. The correct predictions are the sum of true positive and true negative predictions (see Table 4). By contrast in non-classification models, such as regression models, accuracy can be calculated by mean absolute error, mean squared error, root mean squared error, or coefficient of determination (*R*^2^).

Indeed, there is a growing interest in research to identify data-driven biomarkers [83,101]. More recently termed as digital biomarkers, these data-driven indices have unique advantages beyond traditional biomarkers, such as analysis at both the individual and population level, longitudinal and continuous measures, and passive monitoring [83]. More importantly, the emergence and increasing prevalence of wearables with the capability to measure physiological data allows for the further development of putative physiological-based digital biomarkers [101]. These wearables are capable of collecting physiological data, such as blood oxygen saturation, blood pressure, body temperature, electrodermal activity, and heart rate [102]. Not surprisingly, there have been preliminary successes in predicting both physical and mental health using wearable data both through traditional statistical modelling, and more recently, machine learning approaches [103,104,105]. 

In the context of cognitive fatigue, some researchers have recently attempted to predict fatigue levels by adopting the digital biomarker approach. For instance, a study by Al-Libawy and colleagues [106] using data collected from a wrist wearable compared two different machine learning methods (i.e., artificial neural network and support vector machine) to predict cognitive fatigue. Six extracted physiological features were used (i.e., heart rate mean and standard deviation, wrist temperature mean and standard deviations, heart rate and wrist temperature power spectral density), which were chosen based on their influence on classification results. The artificial neural network and support vector machine models achieved 88.3% and 91.3% accuracy in classifying cognitive fatigue state, respectively, though the details of the test sample are unclear and might be inflated by resampling. Furthermore, the models achieved 94.7% and 97.2% accuracy in classifying alertness state, respectively. However, it should be noted that cognitive fatigue and alertness were not directly measured in this study but inferred from the ratio of low- to high-frequency power that was concurrently collected from an electrocardiograph.

Another study using reduced cognitive performance as an index of cognitive fatigue compared the support vector machine and random forest approaches with and without principal components analysis in predicting cognitive fatigue using various HRV features collected with a research-grade electrocardiograph [107]. The three-fold cross-validated random forest model achieved only 57.8% accuracy and, in combination with principal components analysis (leave-one-out cross-validated), improved to 63.9% accuracy. By contrast, they found that the three-fold cross-validated support vector machine model achieved 60% accuracy in predicting cognitive performance. Furthermore, the addition of principal components analysis increased accuracy to 84.4% with a precision of 92.6%, a recall of 73.3%, and an f-score of 81.8%. Notably, some of the selected features for the support vector machine model included not only the ratio of low- to high-frequency power, but also time-domain components, such as the number of R-R intervals, the average of all normalised R–R intervals (AVNN), and the standard deviation of all normalised R–R intervals (SDNN), and non-linear components, such cardiac vagal index (CVI) and cardiac sympathetic index (CSI). These features were measured and averaged from the fifth to eighth trial during the onset of cognitive fatigue. Trial differences between baseline and onset of cognitive fatigue for these features were also included as additional features amongst others. Tsunoda and colleagues [107] highlighted that the use of principal components analysis increased prediction accuracy as the dimensional reduction technique reduced measurement noise. By analysing this model at the individual level, the researchers found that cognitive performance was more accurately predicted in participants with (1) greater number of R-R intervals, (2) larger trial difference in AVNN, (3) larger trial difference in CVI, and (4) larger, but negative, trial difference in the number of R-R intervals. This indicates that cognitive performance can be predicted with higher accuracy in participants with a certain type of physiological profile.

More recently, a study examined various machine learning approaches (i.e., support vector machine, K-nearest neighbour, naive Bayes, and logistic regression) in predicting cognitive fatigue, using data collected from a portable electrocardiogram patch [108]. Using a random forest approach, three time-domain features were selected based on their contribution to prediction accuracy—as indicated by the mean decrease accuracy and mean decrease Gini values: AVNN, the root mean square of the differences between each successive normalised R-R interval (RMSSD), and the proportion of normalised R-R intervals that are more than 50 ms from preceding interval (pNN50). In addition, three frequency-domain features were selected, namely very-low-frequency power, low-frequency power, and total spectral power. After comparing the various machine learning approaches with the combination of up to six of the aforementioned HRV features, this study demonstrated that the K-nearest neighbour model (*k* = 3) with AVNN, low-frequency power, and total spectral power features achieved the highest five-fold cross-validated accuracy of 75.5% in predicting self-reported cognitive fatigue [108], as measured by the Chalder Fatigue Scale [17].

Overall, these studies indicate the feasibility of using machine learning in processing physiological data to monitor cognitive fatigue with moderate to high accuracy rates. It should be highlighted that the studies conducted by Huang and colleagues [108], as well as Tsunoda et al. [107], used electrocardiogram-derived HRV. The biomarker research described in the previous section also predominantly used electrocardiogram to measure HRV [43,44,45,48,49,50,51]. The electrocardiogram uses electrodes to measure the electrical activity of the cardiac cycle [109]. However, most consumer wearables with the capability to measure cardiac activity use photoplethysmography due to its simplicity, comfort, and cost [110,111]. In contrast to electrocardiography, photoplethysmography uses specific wavelengths of light, such as infrared, to measure blood volumetric changes [110], which can be used to estimate blood circulation and associated HRV [112]. Arguably, the peak-to-peak interval observed in photoplethysmography can be interpreted as the equivalent of the R-R intervals of electrocardiography [112]. However, in terms of real-world application, this is only true under non-movement conditions as photoplethysmography recordings are extremely sensitive to motion artefacts, such as wrist movements [112,113,114,115]. For example, a recent study using a clinical-grade electrocardiogram as a benchmark examined several photoplethysmography-based consumer and research-grade wearables under different conditions [113]. In particular, this study reported that wearables had greater measurement error during physical activity than at rest [113]. Within a laboratory setting, Al-Libawy and colleagues [106] have provided indicative evidence of the validity of photoplethysmography-derived features in predicting electrocardiography-derived features. Furthermore, previous biomarker studies have also provided indicative evidence that photoplethysmography-derived frequency-domain features predicted cognitive fatigue [46,47]. Moving forward, future research should examine the reliability and validity of using photoplethysmography in predicting cognitive fatigue beyond laboratory settings. Exploring plausible algorithmic approaches to account for motion artefacts in photoplethysmography would also be imperative within this research area. Prospective researchers should also explore other machine learning methods to predict cognitive fatigue levels to improve accuracy.

## 5. Towards a Biomarker-Informed Machine Learning Model of Cognitive Fatigue

It appears that traditional biomarker research, as well as digital biomarker research, has contributed substantially to our understanding of the physiological features of cognitive fatigue and the degree to which these features can accurately predict varying states of cognitive fatigue. However, it is important to highlight that the traditional biomarker approach predominantly uses statistical modelling, which is viewed as a form of primary data analysis, whereas the digital biomarker approach typically uses data mining, which is considered as a form of secondary data analysis [116]. Due to the ad-hoc nature of data mining, most researchers are very cautious when it comes to the use of data-driven approaches, such as machine learning, as spurious relationships observed within a dataset can be easily misinterpreted [116,117,118,119]. Indeed, such data-driven models are usually atheoretical and thus have limited interpretability [120]. On the flip side, discovering novel relationships in unstructured datasets through data-driven approaches can also help further develop and refine current theoretical accounts [121]. Nonetheless, data-driven models have the potential of achieving high predictive power as their primary goal is to maximise “fit” within a given dataset [117].

In the context of cognitive fatigue, the physiological underpinnings have largely been ignored in data-driven models. As evident in the aforementioned digital biomarker studies in the previous section, machine learning approaches have the potential of producing highly accurate predictive models of cognitive fatigue [106,107,108]. However, the generalisability of these machine learning models should be further evaluated using test datasets. It is also evident in these previous studies that the HRV features selected are fairly inconsistent, which points to the data-driven nature of these models [106,107,108]. Given that the imbalance in sympathetic and parasympathetic nervous activity has been proposed as a physiological correlate of cognitive fatigue, the knowledge derived from traditional biomarker research is particularly informative and should be incorporated into machine learning models to aid development and validation. Specifically, such domain-specific knowledge can help with the selection of parameters, features, or models, which could result in models that are more theoretically coherent, physiologically sound, generalisable, and interpretable [122]. In addition, using multiple biomarkers to build a multivariate model could potentially improve overall predictive power [123]. Thus, adopting a hybridised approach, combining domain-specific knowledge gained from traditional biomarker research with modern machine learning approaches, could potentially help researchers to build a more robust and generalisable model of cognitive fatigue. To this end, future researchers could incorporate our current knowledge on the putative biomarkers of cognitive fatigue (e.g., low-frequency power, high-frequency power, and the ratio of low- to high-frequency power) to aid in feature selection or act as a parameter constraint when developing machine learning models of cognitive fatigue. Open-source tools, such as PySiology, are readily available for researchers to extract these physiological features for machine learning [124]. 

## 6. Issues and Implications

When building a model of cognitive fatigue, potential confounds or closely-related concepts, such as stress and mental workload, should be considered. Stress can be broadly defined as “an emergent process that involves interactions between individual and environmental factors, historical and current events, allostatic states, and psychological and physiological reactivity” [125] (p. 1). Mental workload, in particular, can be viewed as a form of task-related or occupation-related stressor [126]. Mental workload can be objectively defined as the cost of internal resources (i.e., mental effort) to perform at a certain level or complete a task [127,128,129]. Cognitive overloading due to high levels of mental workload may lead to cognitive fatigue [6]. Not surprisingly, previous studies have demonstrated that various frequency-domain HRV indices, such as low-frequency power, high-frequency power, and the ratio of low- to high-frequency power, were associated with increased stress (for review, see [130]). In addition, previous research has shown that the mental with physical workload condition had significantly lower AVNN and lower vagal modulation, indexed by lower pNN50, as compared to the physical only workload condition [131]. Interestingly, Fairclough and colleagues [44] found a significant interaction effect, whereby low-frequency power is higher in the low mental workload condition as compared to the high mental workload condition during the initial period of a 64-min task. While low-frequency power increased in both conditions over time, the increase in low-frequency power was attenuated in the low mental workload condition at the end of the task, resulting in higher levels of low-frequency power in the high mental workload condition than the low mental workload condition [44]. Overall, there appears to be significant overlap and complex interactions amongst cognitive fatigue, mental workload, and stress.

One argument is that putative biomarkers of a particular psychological state should have a certain level of specificity [82,132]. That is, HRV should be more strongly correlated with cognitive fatigue than with stress or mental workload. However, this approach could be erroneous; first, for many individuals, cognitive fatigue, stress, and mental workload could be inherently associated, and thus disentangling these phenomena might not only result in misclassification but could actually be impossible; second, this approach might result in excessive reductionism and thus “paradigm-bound theories” [133]. Hence, prospective researchers should further examine the interaction amongst cognitive fatigue, stress, and mental workload in relation to HRV changes, aiming to represent both “robust reverse inference” (i.e., predicting behaviour from biological responses and predicting biological responses from behaviour). In the context of building models, stress should be accounted for when predicting cognitive fatigue due to the significant overlap in physiological findings between stress and cognitive fatigue. Given the interaction between mental workload and cognitive fatigue on low-frequency power [44], it appears that mental workload is a potential moderator that needs to be considered. Moreover, previous research comparing cognitive fatigue and mental workload levels found that HRV is a better index of cognitive fatigue, whereas heart rate is a better index of mental workload [134]. Hence, future studies could explore using heart rate, as well as heart rate variability, as a measure of mental workload in the development of a more generalisable predictive model of cognitive fatigue, serving as a moderator to account for varying levels of mental workload across different tasks and situations.

## 7. Conclusions and Applications 

Cognitive fatigue is a mental state characterised by the subjective feelings of tiredness, insufficient energy, difficulty with concentration, and impaired ability to think [1,2]. Traditionally, cognitive fatigue has been assessed through self-report and cognitive task performance. Later, biomarker approaches have been adopted to help understand the physiological underpinnings of cognitive fatigue. In particular, the imbalance of sympathetic and parasympathetic nervous activity has been proposed as a physiological correlate of cognitive fatigue. Indeed, as highlighted in the second section of this paper, various HRV indices have been demonstrated to vary as a function of cognitive fatigue levels, indicating that these HRV measures are putative biomarkers of cognitive fatigue. More recently, researchers have also demonstrated that machine learning approaches are capable of predicting cognitive fatigue using physiological data to a high level of accuracy [106,107,108]. Given the ubiquity of wearables that can measure cardiovascular activity, it appears that data collected from these devices have the potential of accurately predicting cognitive fatigue through machine learning approaches. However, the use of domain-specific knowledge from traditional biomarker research with novel machine learning approaches is imperative in building a robust and generalisable predictive model of cognitive fatigue. 

A robust model of cognitive fatigue would allow for the development of a continuous fatigue monitoring system on wearables, which could be used to alert or remind an individual of the need to rest. For instance, given that cognitive fatigue increases the risk of accidents and errors [14,15], such a device may help mitigate not only minor errors but also potentially major accidents. By incorporating fatigue alleviating interventions with this monitoring system, timely and adequate rest could be objectively quantified and maximised. For instance, a previous study has shown that providing HRV biofeedback to participants attenuated cognitive fatigue during a mentally fatiguing task [135]. These effects have also been observed in the chronic fatigue syndrome population, albeit preliminary, where a pilot study has demonstrated that providing HRV biofeedback has improvements on specific cognitive components of fatigue [136]. Besides biofeedback, exposure to natural sounds has also been shown to have a positive effect on cognitive fatigue recovery [137]. Overall, this area of research is important in advancing our knowledge on not only cognitive fatigue monitoring but also cognitive fatigue recovery, which have significant implications in mitigating and minimising the risk of human errors in cognitively fatiguing situations.

## Figures and Tables

**Table 1:** Traditional cognitive fatigue assessment examples.

Method	Indicative Reference	Description	Subjectivity	Disruptiveness	Timeliness	Generalisability
**Self-report**						
Mental Fatigue Scale	[16]	15-items 7-point Likert Assessment of affective, cognitive, and sensory symptoms of fatigue	✓	✓	✕	✓
Chalder Fatigue Scale	[17]	11-items 4-point Likert or Bimodal Assessment of physical and cognitive fatigue	✓	✓	✕	✓
Fatigue State Questionnaire	[18]	4-items 5-point Likert Assessment of physical and cognitive fatigue	✓	✓	✕	✓
**Behavioural Performance**						
Accuracy/Error Rates	[35,38,39,40]	Various cognitive Tasks (e.g., Simon task, Stroop task, Switch Task)	✕	✕	✕	✕
Reaction Time	[33,35,38,39]	Various Cognitive Tasks (e.g., Simon task, Stroop task, Arithmetic Task)	✕	✕	✕	✕
Reaction Time—Intraindividual Variability	[33,39]	Various Cognitive Tasks (e.g., Simon task, Stroop task)	✕	✕	✕	✕

Note. Subjectivity refers to whether the measure is dependent on one’s self-evaluation of his/her level of cognitive fatigue. Disruptiveness refers to whether time is required to be set aside for assessment. Timeliness refers to whether the assessment of the current state of cognitive fatigue can be made in real-time. Generalisability refers to whether the assessments are comparable in other settings, such as different groups, population, task, or situation. Autonomic Nervous System Biomarkers of Cognitive Fatigue.

**Table 2:** Major Heart Rate Variability Indices.

Indices	References	Description	Functional Significance			
			Sympathetic Nervous System	Parasympathetic Nervous System	Overall Autonomic Nervous System	Others
**Time-Domain**						
AVNN	[60]	Average of all normalised R–R intervals				Equivalent to heart rate (inversed)
SDNN	[61]	Standard deviation of all normalised R–R intervals			✓	All cyclic components
RMSSD	[65]	Root mean square of the differences between each successive normalised R-R interval		✓		Respiratory activity
pNN50	[65]	Proportion of normalised R-R intervals that are more than 50 ms from preceding interval		✓		Respiratory activity
**Frequency-Domain**						
Very-low-frequency power	[66,67,68]	Spectral power within the very-low-frequency range of 0.003–0.04 Hz		✓		Thermoregulation; renin-angiotensin-aldosterone activity
Low-frequency power	[65,69,70,71,72]	Spectral power within the low-frequency range of 0.04–0.15 Hz	✓	✓		Baroreflex activity
High-frequency power	[65,70,71]	Spectral power within the high-frequency range of 0.15 to 0.4 Hz		✓		Respiratory activity
Low-frequency/High-frequency power	[70,73]	Ratio of low- to high- frequency spectral power	✓	✓		Sympathovagal balance
Total spectral power	[61]	Spectral power ≤ 0.4 Hz			✓	All cyclic components
**Non-Linear**						
SD1	[74,75,76]	Standard deviation—Poincaré plot (Perpendicular)		✓		Short term changes in heart rate variability
SD2	[75,76,77]	Standard deviation—Poincaré plot (Parallel)	✓	✓		Long term changes in heart rate variability
D_2_	[62,78]	Correlation dimension				Complexity of the system
CVI	[79]	log_10_(longitudinal axis × transverse axis)—Lorenz plot		✓		
CSI	[79]	longitudinal axis/transverse axis—Lorenz plot	✓			

Note. The ticks represent the putative components of the autonomic nervous system each heart rate variability index is thought to reflect. CVI = Cardiac vagal index. CSI = Cardiac sympathetic index.

**Table 3:** Key Performance Metrics of Machine Learning Models.

Metrics	Formula	Description
**Classification Model** [95,99,100]		
Accuracy	$\frac{True\;Positive+True\;Negative}{\begin{array}{c} All\;Cases \end{array}}$	Overall ability of a model to make the correct classification
Precision	$\frac{True\;Positive}{True\;Positive+False\;Positive}$	Ability of a classification model to make correct predictions within the positive class
Sensitivity (Recall)	$\frac{True\;Positive}{True\;Positive+False\;Negative}$	Ability to correctly identity positive labels
Specificity	$\frac{True\;Negative}{True\;Negative+False\;Positive}$	Ability to correctly identity negative labels
F-score	$\frac{2\times Precision\times Sensitivity}{Precision+Sensitivity}$	Harmonic mean of sensitivity and precision
Area Under the Curve (AUC) of the Receiver Operating Characteristic Curve	$\frac{1}{2}\;\left(Sensitivity+Specificity\right)$	Ability of a model to avoid misclassification
**Non-classification models** [96,97,98]		
MAE	$\frac{1}{n}\overset{n}{\underset{i=1}{\sum }}\left|\gamma i-\hat{\gamma }i\right|$	Average of the absolute difference between observed values and predicted values
MSE	$\frac{1}{n}\overset{n}{\underset{i=1}{\sum }}{\left(\gamma i-\hat{\gamma }i\right)}^{2}$	Average of the squared difference between observed values and predicted values
RMSE	$\sqrt{\frac{1}{n}\overset{n}{\underset{i=1}{\sum }}{\left(\gamma i-\hat{\gamma }i\right)}^{2}}$	Standard deviation of the difference between observed values and predicted values
CoD/*R*^2^	$\frac{\sum_{i=1}^{n}{\left(\hat{\gamma }i-\bar{\gamma }\right)}^{2}}{\sum_{i=1}^{n}{\left(\gamma i-\bar{\gamma }\right)}^{2}}$	Proportion of variance in the outcome variable explained by the predictor(s)

Note. MAE = Mean Absolute Error. MSE = Mean Squared Error. RMSE = Root Mean Squared Error.

**Table 4:** Confusion Matrix for Binary Classification Models.

		Actual Classification	
		Positive	Negative
**Predicted Classification**	**Positive**	**True Positive**	**False Positive** **Type I Error**
	**Negative**	**False Negative** **Type II Error**	**True Negative**

Note. The confusion matrix represents the four possible outcomes of a binary classification model.
